# Solid-Phase Extraction Approaches for Improving Oligosaccharide and Small Peptide Identification with Liquid Chromatography-High-Resolution Mass Spectrometry: A Case Study on Proteolyzed Almond Extract

**DOI:** 10.3390/foods11030340

**Published:** 2022-01-25

**Authors:** Yu-Ping Huang, Randall C. Robinson, Fernanda Furlan Goncalves Dias, Juliana Maria Leite Nobrega de Moura Bell, Daniela Barile

**Affiliations:** 1 Department of Food Science and Technology, University of California, Davis, One Shields Avenue, Davis, CA 95616, USA; yphhuang@ucdavis.edu (Y.-P.H.); rcrobinson@ucdavis.edu (R.C.R.); ffgdias@ucdavis.edu (F.F.G.D.); jdemourabell@ucdavis.edu (J.M.L.N.d.M.B.); 2 Department of Biological and Agricultural Engineering, University of California, Davis, One Shields Avenue, Davis, CA 95616, USA; 3 Foods for Health Institute, University of California, Davis, One Shields Avenue, Davis, CA 95616, USA

**Keywords:** peptidomics, glycomics, sample preparation, mixed-mode solid-phase extraction, LC-MS/MS, protein hydrolysates

## Abstract

Reverse-phase solid-phase extraction (SPE) is regularly used for separating and purifying food-derived oligosaccharides and peptides prior to liquid chromatography-tandem mass spectrometry (LC-MS/MS) analysis. However, the diversity in physicochemical properties of peptides may prevent the complete separation of the two types of analytes. Peptides present in the oligosaccharide fraction not only interfere with glycomics analysis but also escape peptidomics analysis. This work evaluated different SPE approaches for improving LC-MS/MS analysis of both oligosaccharides and peptides through testing on peptide standards and a food sample of commercial interest (proteolyzed almond extract). Compared with conventional reverse-phase SPE, mixed-mode SPE (reverse-phase/strong cation exchange) was more effective in retaining small/hydrophilic peptides and capturing them in the high-organic fraction and thus allowed the identification of more oligosaccharides and dipeptides in the proteolyzed almond extract, with satisfactory MS/MS confirmation. Overall, mixed-mode SPE emerged as the ideal method for simultaneously improving the identification of food-derived oligosaccharides and small peptides using LC-MS/MS analysis.

## 1. Introduction

Oligosaccharides are carbohydrates consisting of 2 to 20 monosaccharide units and are widely found in plants and mammalian milk. These non-digestible carbohydrates have been studied for their prebiotic effect on the gut microbiota and their immunomodulatory effects [1,2]. Based on this potential, food products and supplements targeting human gut and digestive health are one of the fastest-growing segments in the food industry, with annual revenue of $39 billion in 2019, which is expected to increase to over $70 billion by 2027 [3]. Similarly, peptides are small fragments of proteins and are universally found in foods. Besides functioning as basic nutrients, peptides with specific structural features can also exhibit bioactivities. Peptides with beneficial activities, such as antimicrobial, antihypertensive, and anti-inflammatory, have been discovered in a wide range of foods [4,5,6].

Enzymatic hydrolysis is considered the preferred method in the food industry for increasing protein extraction yields, enhancing protein digestibility, reducing allergenicity, etc. [7,8,9]. Some peptides generated by enzymatic hydrolysis, have been shown to possess various bioactivities, such that antihypertensive and antibacterial peptides were identified in hypoallergenic infant formula, which had been partially or extensively hydrolyzed [10,11]. Therefore, hydrolysis techniques have been applied to several food products currently on the market [12,13].

The advancement of liquid chromatography-mass spectrometry (LC-MS) and automated data analysis enables the profiling of hundreds of peptides in a sample in only one run and is now widely used in bottom-up proteomics. To avoid ion suppression and ensure data quality in LC-MS analysis, appropriate sample preparation to eliminate interfering substances from complex food materials is indispensable and is regularly fulfilled with reversed-phase solid-phase extraction (SPE) [10,11]. Reverse-phase SPE can separate salts and low-molecular-weight carbohydrates (i.e., simple sugars and oligosaccharides) from peptides because only peptides are retained through the hydrophobic interaction. However, some small peptides, specifically di- and tripeptides, tend to pass through reverse-phase SPE with aqueous eluent and are not recovered in the final peptide eluate [14,15]. 

Peptide identification is most commonly conducted in the context of proteomics studies, which aim to profile the complete set of intact proteins in a sample, and their relative abundances. In bottom-up proteomics, usually only peptides comprising more than four amino acid residues are analyzed for identifying the originating proteins. Focusing the analysis on these longer peptide sequences is done for several practical purposes: since the proteolysis for proteomics is achieved using specific enzymes with well-defined cleavage sites, only limited amounts of smaller peptides are generated. Furthermore, the amino acid sequences of small peptides may be present in many proteins and lack uniqueness, so they are not suitable for verifying the presence of a particular protein. Finally, the algorithms used by MS-based proteomics software often cannot identify di- and tripeptides from tandem-MS data due to the relatively low number of fragment ions generated during fragmentation. In contrast, information about small peptides is significantly valuable for peptidomics, especially for the purpose of studying bioactive peptides. This interested originated from growing evidence showing that several small peptides exert bioactivities and may have a higher chance of surviving digestion as well as entering the blood circulation to exert bioactivity systemically [16,17,18,19]. Moreover, when food material is subject to enzymatic hydrolysis during food processing and then this is followed by the subsequent gastrointestinal digestion after ingestion, it can be expected that proteins will be extensively hydrolyzed and numerous small peptides will be generated. Therefore, small peptides should also be taken into consideration and be characterized when studying the bioactivity of proteolytic products. 

In order to characterize bioactive peptides comprehensively using LC-MS, sample preparation approaches using reverse-phase SPE need to be modified for capturing shorter-length peptides [14,15]. One must also consider that foods often contain both peptides and oligosaccharides, such as milk and plant-based foods. Oligosaccharides can be naturally occurring, generated during processing, or intentionally added as functional ingredients when the foods are lacking such compounds. For food products containing both oligosaccharides and abundant peptides, such as extensively hydrolyzed infant formula, the LC-MS analysis of oligosaccharides will be daunting due to the presence of interfering peptides. In fact, when the peptides are not completely separated from the oligosaccharides, they can cause ion suppression, impede oligosaccharide fragmentation in tandem MS analysis, and consequently diminish identification. A porous graphitized carbon (PGC) column is routinely used for chromatographic separation of oligosaccharides before and during LC-MS [20]. As some peptides strongly bind to PGC sorbent and are very difficult to elute, peptides can cause interferences and even decrease the binding capacity of the PGC column for oligosaccharides, in addition to potentially reducing the column life. Therefore, an effective fractionation of oligosaccharides and peptides would benefit the analysis of both types of analytes.

Incorporating specific binding mechanisms to assist the retention of small and hydrophilic peptides is a potential solution for a more effective fractionation of oligosaccharides and peptides. In theory, protonating peptides’ carboxyl groups through acidification would allow most peptides to carry one or more net positive charge(s) and enable their interaction with cation exchange resins. Mixed-mode SPE, including retention mechanisms of both the reverse phase and strong cation exchange, was used for peptide enrichment prior to LC-MS analysis in a recent study, in which 25 peptides (including 4 tripeptides) were identified from *Bifidobacterium* cultures [21]. Peptide analysis using mixed-mode chromatography has also been reported in a few studies, although C18 reverse phase is still the most popular stationary phase [21,22,23]. However, its application towards the fractionation of oligosaccharides and peptides, especially small peptides, has yet to be evaluated. The objective of this study was to compare different SPE approaches, including mixed-mode (reverse-phase/strong cation exchange) and conventionally used reverse-phase SPE, for their efficacy for fractionating peptides and oligosaccharides and therefore improving peptide and oligosaccharide LC-MS data quality.

## 2. Materials and Methods

### 2.1. Materials

A peptide standard mixture (H2016; containing Gly-Tyr, leucine enkephalin (YGGFL), methionine enkephalin (YGGFM), and angiotensin II (DRVYIHPF)), oligosaccharide standards (raffinose pentahydrate and stachyose hydrate), invertase from baker’s yeast (*S. cerevisiae*), trifluoroacetic acid (TFA), ammonia solution 25% (LC-MS LiChropur), ammonium formate (LC-MS LiChropur), and sodium acetate (molecular biology grade) were obtained from MilliporeSigma (St. Louis, MO, USA). Angiotensin I (DRVYIHPFHL) and neurotensin (pE-LYENKPRRPYIL) were obtained from GenScript (Piscataway, NJ, USA). Acetonitrile (ACN, Optima LC/MS grade), formic acid (Optima LC/MS grade), 50% (*w*/*w*) sodium hydroxide, and a Qubit protein assay kit were purchased from Thermo Fisher Scientific (Waltham, MA, USA). Melibiose and manninotriose were generated from raffinose and stachyose standards, respectively, with the treatment of invertase as described previously [24]. A proteolyzed almond extract was prepared from almond flour, at pilot-scale (~10 L of slurry), as described previously [25]. Briefly, almond flour was extracted with water, and “Neutral Protease 2 million” from *Bacillus subtilis* (BIO-CAT, Virginia, NY, USA), which randomly cleaves peptide bonds in protein structures, was added at an amount equal to 0.5% of the almond flour weight. The extraction was carried out in a 10 L jacketed glass reactor model CG-1965-610M (Chemglass Life Sciences LLC, Vineland, NJ, USA) with a 1:10 solids-to-liquid ratio at 50 °C and pH 9 and stirring at 120 rpm for 60 min. The slurry was separated into four fractions: the insoluble fraction, protein-rich fraction (protein extract), cream, and free oil. The protein extract was used for examining SPE efficacy in this study and was named proteolyzed almond extract. 

### 2.2. Comparison of Procedures for Protein Removal

Ethanol precipitation and ultrafiltration were evaluated for their efficacy in removing proteins in the proteolyzed almond extract. For the precipitation method, 500 μL of the proteolyzed almond extract was mixed with 100 μL cold ethanol and incubated at 4 °C overnight. The mixture was then centrifuged at 4255× *g* at 4 °C for 30 min. The supernatant was separated and dried completely with a centrifugal evaporator at 30 °C and then dissolved with water. For the ultrafiltration method, 500 μL of the proteolyzed almond extract was either filtered directly with 3 kDa molecular weight cut-off (MWCO) centrifugal filter (Amicon, MilliporeSigma) or firstly filtered with 0.2 μm disk filter using a syringe and then filtered sequentially with 10 kDa and 3 kDa MWCO centrifugal filters (Amicon, MilliporeSigma). Centrifugal filtration was conducted at 13,000× *g* at 4 °C for 30 min.

### 2.3. Comparison of Solid-Phase Extraction Approaches

#### 2.3.1. Reverse-Phase Solid-Phase Extraction

Three classic reverse-phase SPE cartridges, including Discovery DSC-18 with either 100 mg sorbent packed in a 1-mL tube (C18 100 mg; MilliporeSigma) or with 500 mg sorbent packed in a 3-mL tube (C18 500 mg; MilliporeSigma), Discovery DSC-8 with 100 mg sorbent packed in a 1-mL tube (C8 100 mg; MilliporeSigma), and a hydrophilic-lipophilic balanced SPE cartridge—Oasis HLB—with 60 mg sorbent packed in a 3-mL tube (HLB 60 mg; Waters, Milford, MA, USA), were tested with the procedures described in Supporting information Table S1. Briefly, the cartridges were conditioned with pure ACN or ACN with either 0.1% TFA or 0.1% formic acid and then accordingly with water, 0.1% TFA in water, or 0.1% formic acid in water. Peptide standard mixtures or supernatants of the proteolyzed almond extract prepared in water, 0.1% TFA, or 0.1% formic acid were loaded to the pre-conditioned SPE cartridges. The cartridges with loaded samples were flushed with three column volumes of water, 0.1% TFA in water, or 0.1% formic acid in water, and then accordingly with three column volumes of 80% ACN or 80% ACN containing either 0.1% TFA or 0.1% formic acid.

#### 2.3.2. Mixed-Mode Solid-Phase Extraction 

Three mixed-mode SPE cartridges comprising reverse-phase and strong cation exchange properties were tested using the procedures listed in the Supporting information Table S2. These included Strata-X-C with 30 mg sorbent packed in a 1-mL tube (X-C 30 mg; Phenomenex, Torrance, CA, USA), Oasis MCX with 30 mg sorbent packed in a 1-mL tube (MCX 30 mg; Waters), and Discovery DSC-MCAX with 100 mg sorbent packed in a 1-mL tube (MCAX 100 mg; MilliporeSigma). For mixed-mode SPE, the cartridges were conditioned with ACN and then with either 0.1% TFA in water or 0.1% formic acid in water. Peptide samples prepared either in 0.1% TFA or 0.1% formic acid were loaded to the cartridges. The cartridges loaded with samples were firstly flushed with 3 mL of 0.1% TFA in water or 0.1% formic acid in water and then flushed with 3 mL of an eluent consisting of 40–50% ACN modified with either 1% ammonia or 250–375 mM ammonium formate. All fractions eluted from SPE cartridges were collected for analysis.

### 2.4. Analysis of Peptide Standards 

Aqueous and high-organic fractions collected from reverse-phase or mixed-mode SPE were analyzed by either a Microflex LRF matrix-assisted laser desorption/ionization-time of flight (MALDI-TOF; Bruker Daltonics, Billerica, MA, USA) mass spectrometer or an Agilent 6520 Accurate-Mass Q-TOF LC-MS with a Chip Cube interface (Agilent Technologies, Santa Clara, CA, USA) to determine the presence of peptide standards in each fraction. For the MALDI-TOF MS analysis, 1 μL of the sample was mixed with 1 μL of α-cyano-4-hydroxycinnamic acid prepared in 30% ACN containing 0.07% TFA. The mixture (0.5 μL) was spotted on a ground steel target plate and dried under vacuum. The analysis was conducted with either linear mode or reflectron mode. Before analyzing the SPE fractions, the instrument was calibrated by the same peptide standard mixtures not subjected to SPE. For the LC-MS analysis, peptide standards were separated on an Agilent Zorbax 300SB-C18 capillary chip with a 40 nL trap (75 μm × 150 mm, 5 μm) at a flow rate of 300 nL min^−1^. The mobile phase consisted of 3% ACN with 0.1% formic acid (*v*/*v*) (A) and 89.9% ACN with 0.1% formic acid (*v*/*v*) (B). The 40-min gradient with linear increase or decrease was programmed as follows: 0–2.3% B from 0.0–0.1 min; 2.3–15% B from 0.1–4.0 min; 15–22% B from 4.0–18.0 min; 22–60% B from 18.0–23.0 min; 60–100% B from 23.0–23.1 min; 100% B from 23.1–28 min; 100–0% B from 28.0–28.1 min; and 0% B from 28.1–40.0 min. Scan ranges were *m*/*z* 70–1800 at 8 spectra sec^−1^ for MS and from *m*/*z* 50–1800 at a precursor abundance dependent speed with a target of 25,000 count spectrum^−1^ for MS/MS. Collision energy (CE; V) of (0.03 × (*m*/*z*) + 2) was used in tandem MS analysis for the top 10 ions in each cycle. The drying gas was set at 325 °C and 5 L min^−1^. A capillary voltage of 1930 V was applied. Detection of peptide standards in the SPE fractions was determined by matching the retention times and the precursor *m*/*z* with the peptide standard mixtures not subjected to SPE.

### 2.5. Measuring the Recovery of Peptides

The efficacy of fractionating peptides and oligosaccharides by reverse-phase and mixed-mode SPE was evaluated with the breakthrough and recovery of peptides in the aqueous fraction and high-organic fraction, respectively, using the proteolyzed almond extract. Aqueous and high-organic fractions of the proteolyzed almond extract were dried with a centrifugal evaporator after collecting from SPE and redissolved with 50 μL of water. The peptide concentration in the redissolved samples was measured by Qubit 4 Fluorometer (Thermo Fisher Scientific) using a Qubit Protein Assay Kit (Thermo Fisher Scientific) according to the manufacturer’s instructions. The breakthrough and recovery were calculated with the following formulas: peptide breakthrough = (total peptides in aqueous fraction/total peptides loaded to SPE) × 100%; peptide recovery = (total peptides in high-organic fraction/total peptides loaded to SPE) × 100%.

### 2.6. Measuring the Recovery of Oligosaccharides 

Oligosaccharides in the aqueous fractions collected from reverse-phase or mixed-mode SPE cartridges using the proteolyzed almond extract were quantified for calculating the recovery of oligosaccharides. The aqueous fractions were directly analyzed after being brought to 5 or 10 mL in a volumetric flask for samples collected from 1 mL or 3 mL SPE cartridges, respectively. The quantification of two oligosaccharides, raffinose and stachyose, was carried out on a Thermo Fisher Dionex ICS-5000+ high-performance anion-exchange chromatography system with a CarboPac PA200 guard column (3 × 50 mm) and a CarboPac PA200 analytical column (3 × 250 mm). The mobile phase was composed of water (A), 200 mM sodium hydroxide (B), and 100 mM sodium hydroxide with 100 mM sodium acetate (C). The analytes were separated by isocratic elution at 25% B at a flow rate of 0.5 mL min^−1^ for 30 min. After the elution of oligosaccharides, the column was regenerated with a linear gradient from 25% B + 0% C to 50% B + 10% C in 5 min, followed by holding at 100% B for 5 min, and equilibrated with 25% B + 0% C for 10 min before the next injection. The oligosaccharides were quantified against calibration curves built with external standards (r^2^ > 0.9995). The recovery was calculated by the formula (oligosaccharides in aqueous fraction/oligosaccharide in the original sample loaded to SPE) × 100%.

### 2.7. Characterization of Oligosaccharides in the Proteolyzed Almond Extract by LC-MS/MS

The aqueous fractions collected from reverse-phase and mixed-mode SPE containing oligosaccharides were further purified by non-porous graphitized carbon SPE (250 mg, 3-mL tube, Supelclean ENVI-Carb, MilliporeSigma). A non-porous graphitized carbon SPE cartridge was conditioned with 80% ACN, equilibrated with water, and then loaded with the aqueous fraction collected from reverse-phase and mixed-mode SPE. The non-porous graphitized carbon SPE cartridge was flushed with three column volumes of water to remove salts and acid. Oligosaccharides were eluted with two column volumes of 40% ACN, dried completely, and redissolved in water. The samples were analyzed with an Agilent 6520 Accurate-Mass Q-TOF LC-MS as described previously [26], with chromatographic separation at a flow rate of 300 nL min^−1^. Oligosaccharides were characterized by examining the MS/MS fragments to determine their monosaccharide composition. Due to potential in-source fragmentation, extracted ion chromatographic peaks of oligosaccharides with various degrees of polymerization that possibly originated from the same oligosaccharide based on their monosaccharide compositions and co-eluted at the same retention time were considered as one identification [24]. Raffinose and stachyose were confirmed by comparing with the corresponding standards. Melibiose and manninotriose were confirmed by comparing with the disaccharide and the trisaccharide generated enzymatically from raffinose and stachyose standards, respectively, with treatment by invertase. 

### 2.8. Characterization of Peptides in the Proteolyzed Almond Extract by LC-MS/MS

Peptide characterization for the proteolyzed almond extract was performed on an Agilent 6520 Accurate-Mass Q-TOF LC-MS. The peptide samples purified by reverse-phase or mixed-mode SPE were injected into an Agilent Zorbax 300SB-C18 chip (40 nL enrichment column, 75 μm × 150 mm; for comparing different protein removal approaches) or an Agilent Polaris-HR-Chip (360 nL enrichment column, 75 μm × 150 mm; for comparing different SPE approaches) and separated by a mobile phase, consisting of 3% ACN with 0.1% formic acid (A) and 89.9% ACN with 0.1% formic acid (B), eluted at a flow rate of 300 nL min^−1^ with the following gradients: 0–2.3% B from 0–0.1 min; 2.3–8% B from 0.1–2.0 min; 8–37% B from 2.0–40.0 min; 37–48 % B from 40.0–45.0 min; 48–100% B from 45.0–45.1 min; 100% B from 45.1–50.0 min; 100–0% B from 50.0–50.1 min; and 0% B from 50.1–65.0 min. The scan range was *m*/*z* 70–1800 for MS and *m*/*z* 50–1800 for MS/MS. The scan speed was set at 8 spectra sec^−1^ for MS and varied with precursor abundance with a target of 25,000 count spectrum^−1^ for MS/MS, respectively. The ESI source was operated on positive mode with a capillary voltage of 1950 V and drying gas at 325 °C and 5 L min^−1^. The top 10 ions with the highest intensities in each cycle were selected for tandem MS analysis with the CE set by a formula of (CE (V) = 0.03 × (*m*/*z*) + 2).

### 2.9. Peptide Data Analysis

Peptide data analysis was performed with PEAKS Studio software (Bioinformatics Solutions Inc., Waterloo, ON, Canada). Medium-sized peptides, defined here as peptides with lengths ≥5 amino acid residues and below the upper limit that generally can be identified by LC-MS/MS (~50 amino acid residues), were identified through database search using the Uniprot database with the species name *Prunus dulcis* (both SwissProt and TrEMBL, accessed 20 June 2019). The mass error tolerance was 10 ppm and 0.02 Da for the precursor and fragment ions, respectively. The enzyme option was set as “None” along with an unspecific digestion mode. A maximum of two variable modifications, including oxidation (M), phosphorylation (STY), and deamidation (NQ), was allowed. The results were filtered with a false discovery rate of 1.0%.

Identification of dipeptides was achieved by de novo sequencing using PEAKS Studio followed by manual MS/MS spectral inspection. The settings for mass error tolerance, enzyme, and digestion mode are the same as those used for database search. A maximum of one variable modification (oxidation (M), phosphorylation (STY), or deamidation (NQ)) was allowed. 

### 2.10. Statistical Analysis

Statistical analyses were performed in R (version 3.5.3). Single-factor analysis of variance and the subsequent pair-wise comparison with the Tukey method (significance level α = 0.05) were conducted to compare the efficacy of different SPE approaches. 

## 3. Results and Discussion 

### 3.1. Efficacy of Different Solid-Phase Extraction Approaches in Binding Peptides 

Although reverse-phase SPE is regularly used in peptide sample preparation, peptides comprising different types and numbers of amino acid residues may possess fairly distinct physicochemical properties (e.g., hydrophobicity and size) and therefore may not be completely recovered with reverse-phase SPE. To understand the retention capability of different SPE cartridges for various peptides, mixtures of peptide standards consisting of 2 to 13 amino acid residues were tested. Peptide standard mixtures were loaded to the pre-conditioned SPE cartridges, which were subsequently washed sequentially with an aqueous eluent and a high-organic eluent. Ideally, oligosaccharides and peptides should be present in the aqueous and high-organic fractions, respectively. 

#### 3.1.1. Reverse-Phase Solid-Phase Extraction

Table 1 presents the effect of acidic modifiers on the recovery of peptide standards from reverse-phase SPE; the aqueous and high-organic fractions were both modified with either 0.1% TFA or 0.1% formic acid. Leucine enkephalin and methionine enkephalin, two endogenous opioid peptide neurotransmitters, were only recovered in the high-organic fraction and not in the aqueous fraction for all the three reverse-phase SPE tested (C18 100 mg, C18 500 mg, and HLB 60 mg), regardless of whether the eluents were modified with acid or not. However, when the eluent was not modified with acid, Gly-Tyr, a dipeptide exerting moderate inhibition against angiotensin-converting enzyme and dipeptidyl peptidase IV [27,28], was only detected in the aqueous fraction. TFA and formic acid increased the retention of Gly-Tyr on reverse-phase SPE, but only “C18 500 mg” flushed with 0.1% TFA in water led to its complete recovery in the high-organic fraction. As Gly-Tyr has a very small molecular size and lacks very hydrophobic side chains, it tended to pass through with aqueous eluent for reverse-phase SPE. When TFA was added to the eluent, the bulky negatively charged trifluoroacetate ions formed ion pairs with Gly-Tyr, which carried a positive charge on the N-terminal amine under a low pH environment, and, therefore, increased the retention of Gly-Tyr. HLB is a hydrophilic-lipophilic balanced copolymer, which provides slight hydrophilic interaction aside from reverse-phase retention and, therefore, was expected to help the retention of less hydrophobic small peptides. However, a complete recovery of Gly-Tyr in the high-organic fraction was not achieved with HLB regardless of the use of acidic modifiers, possibly due to the small size of Gly-Tyr and the relatively weak hydrophilicity of the sorbent.

TFA also improved the recovery of angiotensin II, a vasoconstrictor hormone, in the high-organic fraction from C18 SPE. When the high-organic eluent was not modified with acid, angiotensin II was detected as a tiny peak in the high-organic fractions collected from “C18 100 mg” but not detected in the fraction collected from “C18 500 mg”. The addition of either TFA or formic acid helped the elution of angiotensin II from “C18 100 mg”, whereas only TFA enabled its elution from “C18 500 mg” SPE. In comparison, angiotensin II was successfully recovered by the high-organic eluent from HLB SPE cartridges, even when the eluent was not modified with acid. We hypothesize that residual silanol groups on the C18 sorbent led to the strong retention of angiotensin II, which carries two basic amino acid residues, arginine and histidine. A silanol group can release a proton and consequently carry a negative charge. The acidity of silanol groups varies with types of silanol and is influenced by other factors in sorbent manufacturing [29,30]. The ratio of deprotonated to protonated silanol groups should be higher at a neutral pH than at an acidic pH. Assuming the sorbents of “C18 500 mg” and “C18 100 mg” were identical, the residual silanol groups of “C18 500 mg” should be five times greater than “C18 100 mg”. This could explain the stronger retention of Angiotensin II on the “C18 500 mg” than on the “C18 100 mg”. The addition of acids reduced the deprotonated silanol groups and thus weakened the retention caused by residual silanol groups. Additionally, bulky trifluoroacetate ions further weakened the interaction between deprotonated silanol groups and angiotensin II and therefore enabled the elution of angiotensin II from “C18 500 mg”. In contrast to C18 SPE, HLB SPE is packed with polymerized sorbents and has no silanol group, so the elution of angiotensin II was not affected by the acidic modifiers in the eluent.

#### 3.1.2. Mixed-Mode Solid-Phase Extraction 

Mixed-mode SPE has retention mechanisms of both reverse phase and strong cation exchange. In order to make peptides positively charged, samples must be acidified before being loaded onto the mixed-mode SPE cartridge to protonate both the N-terminal amino group and the C-terminal carboxyl group in peptides. On the other hand, to elute peptides from mixed-mode SPE, either basifying eluent (for deprotonating the N-terminal amino group) or increasing ionic strength of the eluent is necessary. When the peptide standards Gly-Tyr, leucine enkephalin, methionine enkephalin, angiotensin II, and angiotensin I (a precursor to angiotensin II) were applied to “X-C 30 mg”, all five peptides were not detected in the aqueous fraction flushed with an eluent of 0.1% formic acid in water and were exclusively recovered in the high-organic fraction flushed with an eluent containing 80% ACN and 1% ammonia (Table 1 and Table 2). However, neurotensin, a regulatory peptide found in the central nervous system and the gastrointestinal tract, was not recovered in the high-organic fraction using an eluent containing 80% ACN and 1% ammonia (Table 2). We suggest that two arginine and one lysine residues of neurotensin restricted the elution of neurotensin from the mixed-mode SPE. With a pKa of 12.5, the side chain of arginine remained protonated during the elution using an eluent containing 80% ACN and 1% ammonia, which had a measured pH of 10.9. The net charge of neurotensin should be 2+ under these conditions, so neurotensin was still retained by the sulfonyl groups on the mixed-mode sorbent. Interestingly, although angiotensin II and angiotensin I also contain one arginine, the eluent containing 1% ammonia was able to elute the two peptides. To deal with the strong retention of peptides containing multiple arginines, instead of flushing the mixed-mode SPE cartridge with basified eluent, flushing it with an eluent with increased ionic strength by adding ammonium formate as a modifier was tested. The fractions containing ammonium formate could not be analyzed by MALDI-TOF because the ionization of peptides was greatly suppressed by the salts. Instead, as ammonium formate is a volatile salt, the sample could be directly injected into LC-MS without further desalting. The results (Table 2) showed that 250 mM ammonium formate in 50% ACN was able to elute neurotensin from “MCAX 100 mg”, whereas a higher ionic strength of the eluent (375 mM ammonium formate in 40% ACN) was required to elute neurotensin from “X-C 30 mg” and “MCX 30 mg”. We decreased the concentration of ACN in the eluent to increase the ionic strength while avoiding the salt-induced liquid-liquid phase separation. Fortunately, 40–50% ACN still, at least partially, eluted all the peptides tested.

### 3.2. Evaluating Oligosaccharide and Peptide Sample Preparation Approaches Using the Proteolyzed Almond Extract

#### 3.2.1. Comparison of Procedures for Protein Removal 

When analyzing complex food samples, removing proteins before SPE avoids overloading the cartridges and the consequent ineffective binding of target analytes on SPE sorbents. We compared ethanol precipitation and ultrafiltration to evaluate their performance in protein removal using the proteolyzed almond extract. Although ultrafiltration is often used for fractionating peptides based on their sizes, we observed that a significant loss of peptides occurred when filtering the proteolyzed almond extract with a centrifugal filter (MWCO 3 kDa). The extracted ion chromatogram (EIC) peak areas of peptides in the filtrate were much lower than the ones treated by protein precipitation with ethanol (Supporting information Figure S1). The differences in peak area between the samples from filtration and protein precipitation were directly related to the molecular weight of peptides. Even using a sequential filtration with 0.22 μm disk filter, 10 kDa, and 3 kDa, centrifugal filters did not prevent the loss of peptides, so it appeared that membrane fouling caused by insoluble particles and large molecules was not the main factor leading to the loss. Loss of opioid peptides with sizes well below the MWCO using centrifugal filters was also reported in a previous study [31]. The loss might be ascribed to peptide–peptide interaction and peptide aggregation due to the excessively high concentration of peptides on the membrane surface [32,33]. To avoid the risk of losing peptides at the step of protein removal, proteolyzed almond extract that underwent protein precipitation with ethanol was chosen for further studying the efficacy of different SPE approaches in improving the characterization of peptides and oligosaccharides.

#### 3.2.2. Comparison of Solid-Phase Extraction Approaches for Improving Oligosaccharide Characterization 

**Figure 1:**
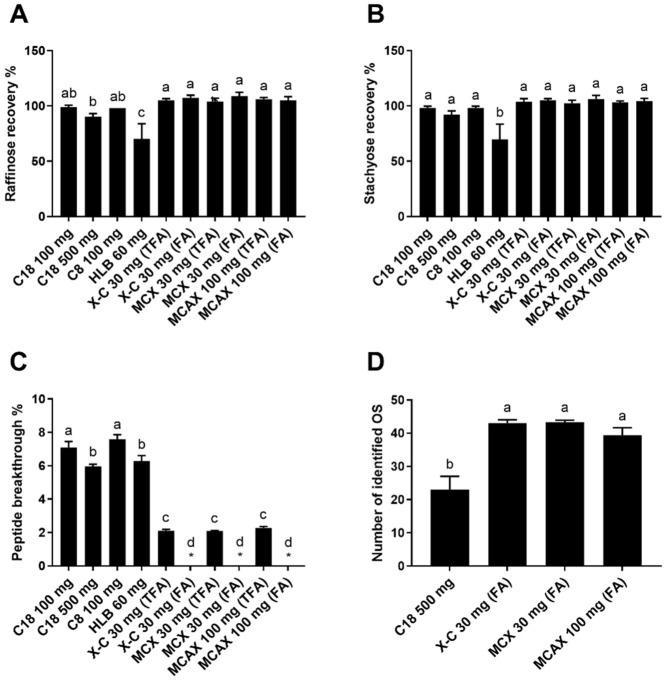
Recovery of raffinose (**A**) and stachyose (**B**), breakthrough of peptides (**C**), and number of oligosaccharides (OS) identified with MS/MS confirmation (**D**) of the aqueous fractions collected from different SPEs loaded with the proteolyzed almond extract. The asterisks indicate cases where the peptide breakthrough was lower than the detection limit (0.8%). Reverse-phase SPE (C18 100 mg, C18 500 mg, C8 100 mg, and HLB 60 mg) was conducted using TFA as a modifier. Mixed-mode SPE cartridges (X-C 30 mg, MCX 30 mg, and MCAX 30 mg) were eluted with either 0.1% TFA in water or 0.1% formic acid (FA) in water. Data are presented as mean ± standard deviation (*n* = 3). Different lowercase letters represent a significant difference at *p* < 0.05.

Oligosaccharides are very hydrophilic compounds due to the abundant hydroxyl groups in their molecular structures. Thus, oligosaccharides are not expected to be retained on hydrophobic SPE sorbents. For mixed-mode SPE, neutral and acidic oligosaccharides are generally uncharged at an acidic pH and are not retained by sulfonyl groups. It is worth noting that oligosaccharides that are positively charged under acidic pH, such as chitosan oligosaccharides, are expected to be retained by sulfonyl groups. Therefore, it is not suitable to use mixed-mode SPE for their purification. To evaluate the effectiveness in fractionating oligosaccharides and peptides with reverse-phase and mixed-mode SPE, we firstly measured the recovery of oligosaccharides from the proteolyzed almond extract. Raffinose and stachyose are two major oligosaccharides in almonds, and the standards are commercially available, so they were chosen for studying the recovery of oligosaccharides. The results showed that a complete or near-complete recovery of the two oligosaccharides was achieved for most SPE cartridges tested except for “HLB 60 mg” (Figure 1A,B). It is likely the cyclic amide providing hydrophilic interaction in the HLB sorbent slightly retained oligosaccharides and reduced their recovery.

A low breakthrough of peptides in the fraction containing oligosaccharides is essential for effective analyses of both oligosaccharides and peptides. Not only would the peptide breakthrough interfere with the analysis of oligosaccharides but also the peptides would never be recovered in the high-organic fraction and therefore would escape characterization. The peptide breakthrough of reverse-phase SPE ranged from 6.0 to 7.6% (Figure 1C). Among the reverse-phase SPE cartridges, peptide breakthroughs of “C18 100 mg” and “C8 100 mg” were significantly higher than those of “C18 500 mg” and “HLB 60 mg”. The lower peptide breakthrough of “C18 500 mg” than “C18 100 mg” indicated that a sufficient sorbent quantity could increase the retention of less hydrophobic peptides. It was also reported previously that the use of underloaded C18 SPE reduced the breakthrough of hydrophilic peptides [34]. The lower breakthrough of “HLB 60 mg” might arise from the higher binding capacity of the polymerized sorbent and the better retention of hydrophilic peptides of HLB sorbent than the silica-based sorbent [34]. Remarkably, the mixed-mode SPE resulted in a much lower peptide breakthrough, which was <0.8% and 2.1–2.3% when eluting with 0.1% formic acid in water and 0.1% TFA in water, respectively (Figure 1C). The low breakthroughs of mixed-mode SPE indicated that the strong cation exchange property played an essential role in peptide retention. Contrary to C18 SPE, modification with 0.1% formic acid gave lower breakthroughs and seemed to be better for retaining small and hydrophilic peptides than with 0.1% TFA. This phenomenon might be caused by the competition between the sulfonyl groups on the mixed-mode sorbents and trifluoroacetate ions in the eluents. When TFA (pKa = 0.52) was used as a modifier, positively charged peptides could also form ion pairs with negatively charged trifluoroacetate ions, aside from being retained by sulfonyl groups. Hydrophobic interaction would therefore become the only retention mechanism for the ion pairs as the peptides’ charge(s) was neutralized. Yet, the ion pairs of very small and hydrophilic peptides were still too polar to be retained by hydrophobic interaction. In contrast, formic acid is a weaker acid (pKa = 3.75), so formic acid molecules in the eluent (pH ~2.6) were mostly undissociated and could not form ion pairs with peptides. Consequently, using TFA as a modifier resulted in higher peptide breakthroughs than using formic acid.

Overall, “C18 500 mg” cartridge gave a satisfactory oligosaccharide recovery and a relatively low peptide breakthrough compared with other reverse-phase SPE cartridges. Therefore, “C18 500 mg” was further compared with mixed-mode SPE on its capability in improving the data quality of oligosaccharide analysis with LC-MS. The oligosaccharide-containing fractions collected from “C18 500 mg” and mixed-mode SPE were subsequently purified with graphitized carbon SPE, a conventional step for oligosaccharide purification, and the oligosaccharides in both samples were analyzed with LC-MS. Several chromatographic peaks corresponding to oligosaccharides comprising hexoses and several peaks corresponding to released N-glycans were identified. We hypothesize that the N-glycans were released from glycopeptides during storage, possibly by glycoamidase originated from almonds as no glycoamidase was added to the proteolyzed almond extract. The reason for the presence of released N-glycans should be further investigated, but it is outside the scope of this study. However, regardless of the reason for the presence of released N-glycans, this diverse oligosaccharide composition is advantageous for our purpose of comparing different SPE approaches in the efficacy of improving oligosaccharide characterization. 

A total of 44 oligosaccharides, including 19 oligosaccharides comprising hexoses and 25 oligosaccharides potentially being released N-glycans, were identified from the aqueous fractions by examining the tandem MS spectra to confirm the monosaccharide compositions (Supporting information Table S3). The identified oligosaccharides may include some anomers, such as the two anomers of melibiose. The number of oligosaccharides identified with tandem MS confirmation for the samples prepared with “X-C 30 mg” and “MCX 30 mg” was near twice the number obtained when using “C18 500 mg” (Figure 1D). This result indicates that the mixed-mode SPE may more effectively remove interferences and significantly improve the results of oligosaccharide analysis using LC-MS.

#### 3.2.3. Comparison of Solid-Phase Extraction Approaches for Improving Peptide Characterization

##### Medium-Sized Peptides 

**Figure 2:**
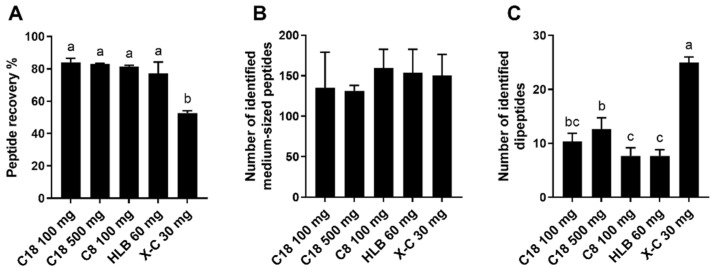
Recovery of proteins/peptides in high-organic fraction (**A**) and numbers of medium-sized peptides (peptide length ≥5 amino acid residues); (**B**) and dipeptides (**C**) identified by LC-MS from the proteolyzed almond extract collected from different SPE techniques. Reverse-phase SPE (C18 100 mg, C18 500 mg, C8 100 mg, and HLB 60 mg) was conducted using TFA as a modifier. Mixed-mode SPE (X-C-30 mg) was washed with 0.1% formic acid (aqueous fraction) and eluted with 80% ACN containing 1% ammonia for recovering peptides (high-organic fraction). Data are presented as mean ± standard deviation (*n* = 3). Different lowercase letters represent a significant difference at *p* < 0.05.

After loading the deproteinized proteolyzed almond extract to the reverse-phase and mixed-mode SPE cartridges and eluting the fraction containing oligosaccharides, a high-organic solvent was used for peptides elution. The peptide recovery of reverse-phase SPE ranged between 77 and 84%, which was higher than that of “X-C 30 mg” eluted with 80% ACN and 1% ammonia (53% recovery) (Figure 2A). The lower recovery for the “X-C 30 mg” could be explained by the loss of peptides containing multiple arginine and lysine residues due to the strong retention by sulfonyl groups on the mixed-mode sorbent, as already observed with neurotensin. The comparison of the results for peptides identified by database search in the various samples prepared by employing different SPE approaches revealed that peptides containing multiple basic amino acids, such as arginine residues (e.g., peptide with sequence LDFVQPP**R**G**R)**, and peptides containing one arginine along with multiple lysine residues (e.g., VTVP**K**EEE**KR**PQV**K)** were exclusively identified and detected in the samples prepared with reverse-phase SPE. Additionally, peptides containing multiple lysine residues, such as IMD**K**I**K**E**K**LPGQH, were only partially recovered in the samples prepared with “X-C 30 mg” due to the strong retention by sulfonyl groups, as evidenced by the smaller peak areas than the reverse-phase SPE samples. In comparison, peptides containing only one arginine or one lysine residue, such as LDFVQPP**R**, had comparable peak areas in the “X-C 30 mg” and all the reverse-phase SPE samples, which was in agreement with our prior results obtained by testing peptide standards. 

Despite the failure to identify some peptides containing multiple arginine and lysine residues in the “X-C 30 mg” high-organic fraction, due to the poor elution from the SPE, similar numbers of peptide sequences were overall obtained by database search (peptide length ≥5 amino acid residues) in the samples of “X-C 30 mg” and all the reverse-phase SPE methods tested (Figure 2B). This means that in reality peptides with numerous arginine and lysine residues likely accounted for an insignificant portion of all the identified peptides. However, due to the potential of unsuccessful recovery of peptides containing multiple basic amino acid residues, one must consider the sample types and the analytes of interest when selecting SPE approaches. For example, mixed-mode SPE can be used for tryptic peptides that generally contain only one arginine or one lysine, as demonstrated in a previous study [21]. In contrast, mixed-mode SPE is not ideal when attempting to identify specifically cationic antimicrobial peptides, which typically contain several basic amino acid residues [35].

In an effort to improve the recovery of peptides containing multiple basic amino acid residues, we tried eluting peptides from “X-C 30 mg” using an eluent with increased ionic strength (40% ACN, 400 mM ammonium formate), but we were not able to measure the peptide recovery because ammonium formate caused a severe interference with the Qubit assay. Therefore, the samples eluted by 40% ACN and 400 mM ammonium formate were directly analyzed with LC-MS. Although the peptides containing two arginine residues were now successfully detected by LC-MS, ammonium formate in the final sample caused peak shape broadening and tailing for many peptides. Flushing the loading column with a larger volume of mobile phase during sample injection helped eliminate ammonium formate; however, as a consequence, the small and hydrophilic peptides were lost, thus eliminating a major goal of using mixed-mode SPE. 

##### Small Peptides 

Peptides with four or fewer amino acid residues can be identified from mass spectral data by de novo identification. In the present study, we focused on dipeptides because it is generally more challenging to retain them by SPE with hydrophobic interactions than tri- and tetrapeptides. We were able to identify 30 dipeptides in the proteolyzed almond extract. All the 30 dipeptides were identified in the samples prepared with “X-C 30 mg”, eluted with 80% ACN and 1% NH_3_, with satisfying MS/MS confirmation, whereas the numbers were substantially lower for the samples prepared with reverse-phase SPE (9–17 dipeptides) (Table 3 and Figure 2C). Several of the dipeptides exclusively identified in the samples prepared by “X-C 30 mg” were early-eluting peptides comprised of hydrophilic amino acid residues, such as glutamine. The improved retention of hydrophilic dipeptides achieved with “X-C 30 mg” can be attributed to the strong cation exchange property of the sorbent. The analysis of small peptides has recently been recognized as a major challenge in MS-based analysis [36,37]. Even though this is due to the convergence of many factors (including sample preparation, peptide enrichment, and data analysis), recovering small peptides using an appropriate sample preparation strategy is one of the critical steps that can help overcome such hurdle. This study proposed the feasibility of using mixed-mode SPE as a strategy to recover small peptides and demonstrated its success in enabling further analysis of small peptides by LC-MS.

## 4. Conclusions

Disorders caused by dysregulated gastrointestinal microbiomes are increasingly common. Currently, such disorders are treated by small-molecule antimicrobial drugs, which unfortunately lack selectivity, killing both pathogenic and commensal organisms and thus leading to further disruption of the microbiome. Therefore, there is growing interest in modulating gut health with novel food products rich in functional ingredients such as peptides and oligosaccharides, which have significant potential to impact human health. In order to comprehensively characterize small and medium-sized bioactive peptides and oligosaccharides in foods using LC-MS, sample preparation approaches using various SPE need to be adapted for capturing all compounds of interest. The proteolyzed almond extract was selected as a model because almond proteins contain a high proportion of hydrophilic amino acids, resulting in a more difficult peptide recovery via conventional reverse-phase SPE. Therefore, having established a successful model system on a challenging food material, these findings could be universally applied to other abundant matrices such as animal proteins, which are known to contain more hydrophobic amino acid residues. Based on the evaluation in this study, when studying proteolyzed food samples, in which oligosaccharides would be found together with abundant peptides, mixed-mode SPE should be preferred over reverse-phase SPE because it led to lower peptide breakthrough and therefore improved oligosaccharide identification validated by tandem MS confirmation. When the purpose of characterization is mainly focused on the discovery of bioactive peptides, factors such as peptide size, hydrophobicity, and charge must be taken into account. For peptides with sufficient hydrophobicity (which are generally medium-sized peptides), C18 SPE with an adequate amount of sorbent leads to more robust results. Although mixed-mode SPE could render a similar number of medium-sized peptides as reverse-phase SPE, failure to identify peptides containing multiple basic amino acid residues might be a concern. Nevertheless, when small and hydrophilic peptides are of interest, mixed-mode SPE remains the ideal choice because of its effective retention of these types of peptides. In summary, this study compared the efficacy of separating oligosaccharides and peptides with reverse-phase and mixed-mode SPE approaches, providing a useful guide for selecting specific sorbents and solvents based on the properties of food samples and compounds of interest. 

## Figures and Tables

**Table 1:** Detection of peptide standards in aqueous (aq) and high-organic (org) fractions collected from different solid-phase extraction techniques.

Solid-Phase Extraction	Glycyl Tyrosine		Leucine Enkephalin (YGGFL)		Methionine Enkephalin (YGGFM)		Angiotensin II (DRVYIHPF)	
	aq	org	aq	org	aq	org	aq	org
*no modifier*								
C18 100 mg	✔ ^1^			✔		✔		✔ (low)
C18 500 mg	✔			✔		✔		
HLB 60 mg	✔			✔		✔		✔
*FA* ^2^ *as modifier*								
C18 100 mg	✔			✔		✔		✔
C18 500 mg	✔	✔		✔		✔		
HLB 60 mg	✔	✔		✔		✔		✔
*TFA as modifier*								
C18 100 mg	✔	✔		✔		✔		✔
C18 500 mg		✔		✔		✔		✔
HLB 60 mg	✔	✔		✔		✔		✔
*FA as modifier for aq; NH_3_ as modifier for org*								
X-C 30 mg		✔		✔		✔		✔

^1^ Checkmark represents the detection of the peptide by MALDI-TOF MS or LC-QTOF MS from the corresponding fraction. ^2^ FA, formic acid; TFA, trifluoroacetic acid.

**Table 2:** Detection of peptide standards in high-organic fraction collected from mixed-mode solid-phase extraction ^1^ eluted by eluents modified by ammonia or ammonium formate.

Solid-Phase Extraction	Composition of High-Organic Eluent	Angiotensin I	Neurotensin
*NH_3_ as modifier*			
X-C 30 mg	80% ACN, 1% NH_3_	✔ ^2^	
MCX 30 mg	80% ACN, 1% NH_3_	✔	
MCAX 100 mg	80% ACN, 1% NH_3_	✔	
*NH4COOH as modifier*			
X-C 30 mg	50% ACN, 250 mM NH_4_COOH	✔	
MCX 30 mg	50% ACN, 250 mM NH_4_COOH		
MCAX 100 mg	50% ACN, 250 mM NH_4_COOH	✔	✔
X-C 30 mg	40% ACN, 375 mM NH_4_COOH	✔	✔
MCX 30 mg	40% ACN, 375 mM NH_4_COOH	✔	✔
MCAX 100 mg	40% ACN, 375 mM NH_4_COOH	✔	✔

^1^ All the mixed-mode SPE were washed with 0.1% formic acid before eluting high-organic fraction. ^2^ Checkmark represents the detection of the peptide by MALDI-TOF MS or Q-TOF LC-MS from the corresponding fraction.

**Table 3:** Dipeptides identified with LC-MS/MS in the high-organic fractions, of the proteolyzed almond extract, prepared with each solid-phase extraction technique ^1^.

Peptide Sequence	Retention Time (min)	C18 100 mg	C18 500 mg	C8 100 mg	HLB 60 mg	X-C 30 mg
Gln-Gln	2.00					✔ ^2^
Gly-Gln	2.00					✔
Ala-Pro	2.21					✔
Gly-Val	2.28					✔
Lxx-Glu ^3^	3.24					✔
Ser-Tyr	3.58		✔			✔
Gly-Tyr	3.65		✔			✔
Val-Pro	3.84		✔			✔
Thr-Tyr	3.93					✔
Ser-Lxx	4.80					✔
Gly-Lxx	5.13		✔			✔
Ala-Lxx	5.23					✔
Thr-Lxx	6.17		✔			✔
Val-Tyr	6.28	✔	✔			✔
Lxx-Val	6.83	✔				✔
Ser-Phe	6.91					✔
Gly-Phe	7.12	✔	✔		✔	✔
Ala-Phe	7.27					✔
Lxx-Pro	7.38	✔	✔			✔
Val-Lxx	7.66		✔			✔
Phe-Pro	9.45	✔	✔	✔	✔	✔
Lxx-Phe	10.42	✔		✔		✔
Trp-Pro	11.08	✔	✔	✔	✔	✔
Tyr-Trp	11.72					✔
Val-Met	11.74	✔	✔	✔	✔	✔
Lxx-Phe	11.88	✔	✔	✔	✔	✔
Lxx-Trp	12.75	✔	✔	✔	✔	✔
Lxx-Trp	13.39	✔	✔	✔	✔	✔
Phe-Phe	13.40	✔	✔	✔	✔	✔
Phe-Trp	15.06	✔	✔	✔	✔	✔

^1^ Reverse-phase SPE (C18 100 mg, C18 500 mg, C8 100 mg, and HLB 60 mg) was eluted with 0.1% TFA (aqueous fraction) followed by 80% ACN modified with 0.1% TFA (high-organic fraction). Mixed-mode SPE (X-C 30 mg) was eluted with 0.1% formic acid (aqueous fraction), and 80% ACN was modified with 1% NH_3_ (high-organic fraction). ^2^ Checkmarks represent peptide identification, with MS/MS confirmation in at least one of the three replicates. ^3^ Lxx denotes that the amino acid residue could be either Leu or Ile.

## Data Availability

The data presented in this study are available in article and supplementary material.

## Supplementary Materials

The following supporting information can be downloaded at: https://www.mdpi.com/article/10.3390/foods11030340/s1, Figure S1: Extracted ion chromatograms (EIC) of selected peptides from the proteolyzed almond extract purified with different protein removal approaches; Table S1: Procedures of different reverse-phase solid-phase extraction techniques; Table S2: Procedures of different mixed-mode solid-phase extraction techniques; Table S3: List of oligosaccharides identified from the proteolyzed almond extract with LC-MS.
