# Supplementary material for: Solid-Phase Extraction Approaches for Improving Oligosaccharide and Small Peptide Identification with Liquid Chromatography-High-Resolution Mass Spectrometry: A Case Study on Proteolyzed Almond Extract
Source: Foods. 2022 Jan 25;11(3):340. doi: 10.3390/foods11030340 (PMC8834518; doi:10.3390/foods11030340)
Supplement: Supplementary file 1 [file foods-11-00340-s001.zip › foods-1543829-supplementary.pdf]

Table S1. Procedures of different reverse-phase solid-phase extraction techniques.

solid-phase extraction step	no modifier				TFA[1] as modifier				FA as modifier			
	C18 100 mg	C18 500 mg	C8 100 mg	HLB 60 mg	C18 100 mg	C18 500 mg	C8 100 mg	HLB 60 mg	C18 100 mg	C18 500 mg	C8 100 mg	HLB 60 mg
condition	3 column volumes of ACN				3 column volumes of ACN with 0.1 % TFA				3 column volumes of ACN with 0.1 % FA			
equilibration	3 column volumes of water				3 column volumes of water with 0.1 % TFA				3 column volumes of water with 0.1 % FA			
sample loading	samples prepared in water				samples prepared in 0.1% TFA				samples prepared in 0.1% FA			
oligosaccharide elution (aq)	3 column volumes of water				3 column volumes of 0.1% TFA				3 column volumes of 0.1% FA			
peptide elution (org)	3 column volumes of 80% ACN				3 column volumes of 80% ACN with 0.1% TFA				3 column volumes of 80% ACN with 0.1% FA			

[1] TFA, trifluoroacetic acid; FA, formic acid; ACN, acetonitrile; aq, aqueous fraction; org, high-organic fraction.

Table S2. Procedures of different mixed-mode solid-phase extraction techniques.

solid-phase extraction step	FA[1] and NH_3 as modifiers			TFA and NH_3 as modifiers			FA and NH_4COOH as modifiers		
	X-C 30 mg	MCX 30 mg	MCAX 100 mg	X-C 30 mg	MCX 30 mg	MCAX 100 mg	X-C 30 mg	MCX 30 mg	MCAX 100 mg
condition	3 column volumes of ACN			3 column volumes of ACN			3 column volumes of ACN		
equilibration	3 column volumes of water with 0.1% FA			3 column volumes of water with 0.1% TFA			3 column volumes of water with 0.1% FA		
sample loading	samples prepared in 0.1% FA			samples prepared in 0.1% TFA			samples prepared in 0.1% FA		
oligosaccharide elution (aq)	3 column volumes of 0.1% FA			3 column volumes of 0.1% TFA			3 column volumes of 0.1% FA		
peptide elution (org)	3 column volumes of 80% ACN with 1% NH_3			3 column volumes of 80% ACN with 1% NH_3			3 column volumes of 50% ACN with 250 mM NH_4COOH or 40% ACN with 375 mM NH_4COOH		

[1] FA, formic acid; TFA, trifluoroacetic acid; ACN, acetonitrile; aq, aqueous fraction; org, high-organic fraction.

Table S3. List of oligosaccharides identified from the proteolyzed almond extract with LC-MS

monosaccharide composition[1]	retention time (min)	observed MS1 ions
Hex$_2$ (melibiose)[2]	0.7	325, 685, 343, 667, 487, 649, 505
Hex$_2$ (melibiose)	1.2	325, 685, 343, 667, 487, 505, 649
Hex$_3$ (manninotriose)	3.2	325, 487
Hex$_3$	4.0	505, 343
Hex$_3$ (manninotriose)	4.6	325, 487
Hex$_2$HexNAc$_2$Fuc$_1$	8.0	895
Hex$_4$	8.5	667
Hex$_3$ (raffinose)	9.4	325, 343, 685, 667, 505, 487
Hex$_3$	10.7	505, 829
Hex$_3$HexNAc$_4$Fuc$_1$Xyl$_1$	10.8	798
Hex$_4$HexNAc$_2$Fuc1Xyl$_1$	10.8	1351, 676
Hex$_3$HexNAc$_2$Fuc1Xyl$_1$	11.0	1189, 1043
Hex$_4$ (stachyose)	11.3	325, 487, 505, 343
Hex$_4$HexNAc$_4$Fuc$_2$Xyl$_1$	12.4	952
Hex$_4$HexNAc$_4$Fuc$_1$Xyl$_1$	12.4	879
Hex$_3$HexNAc$_3$Fuc$_1$Xyl$_1$	12.5	696
Hex$_4$HexNAc$_4$Fuc$_1$Xyl$_1$	13.1	879
Hex$_4$HexNAc$_4$Fuc$_2$Xyl$_1$	13.2	952

$Hex_2HexNAc_2Fuc_1Xyl_1$	13.3	1027
$Hex_3HexNAc_4Fuc_1Xyl_1$	13.7	798
Hex_4	14.1	649, 667, 487, 325, 505
$Hex_5HexNAc_4Fuc_3Xyl_1$	14.5	1106
$Hex_3HexNAc_2Fuc_1Xyl_1$	14.7	1189, 505, 487, 829
$Hex_2HexNAc_2Xyl_1$	14.9	881
$Hex_3HexNAc_4Xyl_1$	15.0	725
Hex_4	15.2	649, 667
Hex_4	15.5	649, 487, 325
$Hex_3HexNAc_4$	15.9	659
$Hex_3HexNAc_4Xyl_1$	16.3	725
Hex_4	16.4	505, 325, 487, 343, 667
$Hex_3HexNAc_2Xyl_1$	16.4	1043
$Hex_3HexNAc_2$	16.6	911
$Hex_2HexNAc_2Xyl_1$	16.7	881
$Hex_5HexNAc_4Fuc_3Xyl_1$	17.4	1106
$Hex_3HexNAc_2Xyl_1$	18.3	1043
$Hex_3HexNAc_2$	18.4	911
$Hex_3HexNAc_2Xyl_1$	18.9	1043
$Hex_5HexNAc_4Fuc_2Xyl_1$	20.1	1033
Hex_4	25.8	667, 649, 325, 487
Hex_5	26.0	829
Hex_4	26.1	667, 649, 487
Hex_4	26.4	667, 649, 487
Hex_5	26.6	829
Hex_5	30.6	829, 649

[1] Abbreviation for monosaccharides: Hex, hexose; HexNAc, N-acetylhexosamine; Fuc: fucose; Xyl: xylose. [2] Melibiose and manninotriose were confirmed by comparing with melibiose and manninotriose generated enzymatically from raffinose and stachyose standards, respectively, with treatment by invertase. Raffinose and stachyose were confirmed by comparing with the corresponding standards.

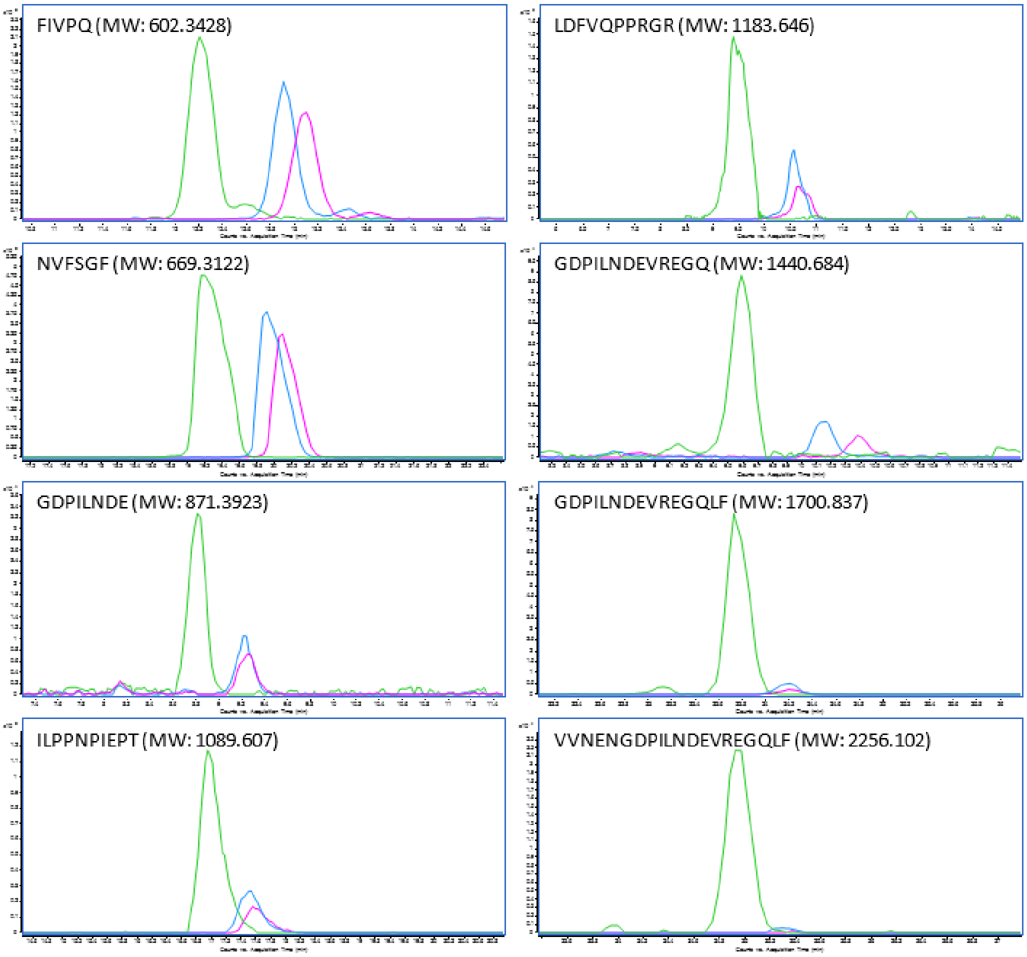

Figure S1. Extracted ion chromatograms (EIC) of selected peptides from the proteolyzed almond extract purified with different protein removal approaches (green line: protein precipitation; blue line: centrifugal filtration (MWCO 3,000 Da); pink line: sequential filtration with disk filter (0.22 µm) and centrifugal filter (MWCO 10,000 Da and then MWCO 3,000 Da)) followed by “C18 500 mg” SPE (TFA as modifier).
